# 2D WSe_2_ Flakes for Synergistic Modulation of Grain Growth and Charge Transfer in Tin‐Based Perovskite Solar Cells

**DOI:** 10.1002/advs.202004315

**Published:** 2021-03-27

**Authors:** Tianyue Wang, Fangyuan Zheng, Guanqi Tang, Jiupeng Cao, Peng You, Jiong Zhao, Feng Yan

**Affiliations:** ^1^ Department of Applied Physics The Hong Kong Polytechnic University Hung Hom Kowloon 999077 Hong Kong

**Keywords:** 2D transition‐metal dichalcogenides, charge transfer, grain growth, tin‐based perovskite solar cells, WSe_2_

## Abstract

Tin (Sn)‐based perovskites with favorable optoelectronic properties and ideal bandgaps have emerged as promising alternatives to toxic lead (Pb)‐based perovskites for photovoltaic applications. However, it is challenging to obtain high‐quality Sn‐based perovskite films by solution process. Here, liquid‐exfoliated 2D transition‐metal dichalcogenides (i.e., MoS_2_, WS_2_, and WSe_2_) with smooth and defect‐free surfaces are applied as growth templates for spin‐coated FASnI_3_ perovskite films, leading to van der Waals epitaxial growth of perovskite grains with a growth orientation along (100). The authors find that WSe_2_ has better energy alignment with FASnI_3_ than MoS_2_ and WS_2_ and results in a cascade band structure in resultant perovskite solar cells (PSCs), which can facilitate hole extraction and suppress interfacial charge recombination in the devices. The WSe_2_‐modified PSCs show a power conversion efficiency up to 10.47%, which is among the highest efficiency of FASnI_3_‐based PSCs. The appealing solution phase epitaxial growth of FASnI_3_ perovskite on 2D WSe_2_ flakes is expected to find broad applications in optoelectronic devices.

## Graphical abstract

Liquid‐exfoliated 2D transition‐metal dichalcogenides (MoS_2_, WS_2_, and WSe_2_) are introduced as growth templates for spin‐coated FASnI_3_ perovskite films, leading to van der Waals epitaxial growth of perovskite grains with a growth orientation along (100). The good energy alignment in the NiO*_x_*/WSe_2_/FASnI_3_ structure facilitates hole extraction and suppresses interfacial charge recombination, leading to the best PCE of 10.47% for the PSC.

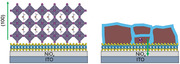

Organic–inorganic hybrid halide perovskite solar cells (PSCs) have shown rapid progress in power conversion efficiency (PCE) in the past ten years, which presently has exceeded 25%.^[^ ^1^, ^2^, ^3^ ^]^ However, the top‐performing PSCs are based on toxic lead halide perovskite materials, which are detrimental to the environment. Sn‐based perovskites have been recognized as ideal alternatives due to their favorable optoelectronic properties and environmentally friendly characters.^[^ ^4^, ^5^, ^6^, ^7^ ^]^ Notably, Sn‐based PSCs exhibit the highest performance among the categories of Pb‐free PSCs, while their performance still lags far behind that of Pb‐based counterparts.^[^ ^8^, ^9^ ^]^

The inferior performance of Sn‐based PSCs can be ascribed to the following reasons. One critical factor is the poor quality Sn‐based perovskite films with rough surface and high‐density of traps.^[^ ^10^, ^11^ ^]^ To achieve high‐performance Sn‐based PSCs, a uniform and continuous perovskite film with large grain sizes and a preferred orientation is required. Several strategies have been developed to modulate the crystallization of Sn‐based perovskites.^[^ ^12^, ^13^, ^14^ ^]^ For example, Meng et al. reported the introduction of pentafluorophen‐oxyethylammonium iodide (FOEI) into the precursor solution for surface‐controlled growth of FASnI_3_ perovskites.^[^ ^15^ ^]^ Yu et al. tuned MA*_x_*FA_1−_ *_x_*SnI_3−_ *_x_*Br*_x_* perovskites with preferential facet of (001) via compositional engineering.^[^ ^16^ ^]^ Qiu et al. introduced *n*‐butylamine (BA) and phenylethylamine (PEA) as the intermediate phase suppressor to obtain uniform 2D Ruddlesden–Popper Sn‐based perovskites with ordered crystal nucleation.^[^ ^17^ ^]^ More recently, the solution of *n*‐propylammonium iodide (PAI) was utilized to induce the recrystallization and templated growth of FASnI_3_.^[^ ^18^ ^]^ Despite the success in modulating the nucleation and crystallization of Sn‐based perovskites, the obtained perovskite films cannot meet the requirement for high‐performance PSCs due to the easy oxidation of Sn^2+^ to Sn^4+^ that can induce high levels of p‐type doping in perovskite films. Thus reducing agents should be introduced to prohibit the oxidation of the perovskite films,^[^ ^19^, ^20^, ^21^ ^]^ which in turn leads to a less conductive layer unfavorable for charge transfer on grain surfaces.^[^ ^22^, ^23^, ^24^ ^]^

Another important factor is the interfacial energy loss that limits the device PCE. The interface states and energy level mismatching between Sn‐based perovskites and common charge transport materials can induce severe non‐radiative recombination and result in low open‐circuit voltage (*V*_oc_) and fill factor (FF).^[^ ^25^, ^26^ ^]^ To solve this problem, Jokar et al. introduced guanidinium (GA) cation in the FA/GA mixed perovskite to match its band structure with PEDOT:PSS and C_60_.^[^ ^27^ ^]^ Thereafter, Nishimura et al. tuned the band structure of GeI_2_‐doped (FA_1−_ *_x_*EA*_x_*)_0.98_EDA_0.01_SnI_3_ perovskite by introducing ethylammonium cation (EA) with different amount to match the energy levels of the transport layers.^[^ ^28^ ^]^ Very recently, Jiang et al. substituted [6,6]‐phenyl‐C_61_‐butyric acid methyl ester (PCBM) with indene‐C_60_ bisadduct (ICBA) as an electron transport material, which shows a smaller energy level offset with 2D perovskite component due to a higher lowest unoccupied molecular orbital (LUMO) level of the latter.^[^ ^29^ ^]^ In addition, the energy loss in Sn‐based PSCs can be reduced by interface optimization of hole transport layers (HTLs).^[^ ^30^, ^31^ ^]^

2D semiconductors, for example, transition‐metal dichalcogenides, demonstrate unique optoelectronic properties such as high carrier mobilities, tunable band structures, and optical transparency,^[^ ^32^, ^33^ ^]^ making them excellent interlayer materials in PSCs.^[^ ^34^, ^35^ ^]^ In the present work, we introduced liquid‐phase‐exfoliated few‐layer MoS_2_, WS_2_, and WSe_2_ (the formula can be referred as *MX*_2_, where *M* is a transition metal and *X* is a chalcogenide) flakes between NiO*_x_* HTL and FASnI_3_ perovskite for achieving high performance inverted PSCs. *MX*_2_ is utilized as a growth template for preparing epitaxial Sn‐based perovskite films by spin coating. Perovskite films on *MX*_2_ exhibit (011) and (01$\bar{1}$) oriented growth along the substrate and out‐of‐plane growth with preferred (100) orientation. We find that the higher valence band maximum (VBM) of WSe_2_ than MoS_2_ and WS_2_ matches well with that of FASnI_3_ perovskite, which promotes cascade hole extraction at the interface and suppresses interfacial charge recombination, and the highest PCE of 10.47% is achieved in WSe_2_‐incorporated devices.

Several types of *MX*_2_ (i.e., MoS_2_, WS_2_, and WSe_2_) flakes were prepared by liquid phase exfoliation of their bulk powders, as schematically shown in **Figure** **1**a. In brief, 100 mg of the corresponding material powders were added to 10 ml isopropyl alcohol (IPA) in centrifuge tubes and then sonicated for 12 h in an ice bath. The dispersions were then centrifuged to take the supernatant containing exfoliated flakes. The exfoliated flake samples were characterized with ultraviolet–visible (UV–vis) and Raman spectroscopies. Figure 1b shows the image (inset) and Tauc plots of the *MX*_2_ flakes in IPA dispersions. The optical bandgaps of MoS_2_, WS_2_, and WSe_2_ flakes are estimated to be ≈1.70, ≈1.77, and ≈1.48 eV, respectively, which are consistent with the values reported in literature.^[^ ^36^, ^37^ ^]^ Figure 1c shows the Raman spectra of *MX*_2_ flakes spin‐coated on Si wafers. Strong characteristic peaks at around 381 and 405 cm^−1^ for MoS_2_, 350 and 415 cm^−1^ for WS_2_, and 250 cm^−1^ for WSe_2_ can be observed, which correspond to the E_2g_^1^and A_1g_ modes of these *MX*_2_ with a few layer thickness.^[^ ^38^, ^39^ ^]^ Such *MX*_2_ flakes are typically thin nanosheets with the thickness in the 5–7 nm range, as revealed by the transmission electron microscopy (TEM) (Figure 1d–f) and atomic force microscopy (AFM) (Figure S1, Supporting Information). The insets in Figure 1d–f show the HRTEM images of the corresponding *MX*_2_ flakes. A clear lattice fringe of 0.27, 0.27, and 0.28 nm are observed, corresponding to the (100) plane distances of MoS_2_ (PDF#65‐0160), WS_2_ (PDF#08‐0237), and WSe_2_ (PDF#38‐1388), respectively.

**Figure 1:**
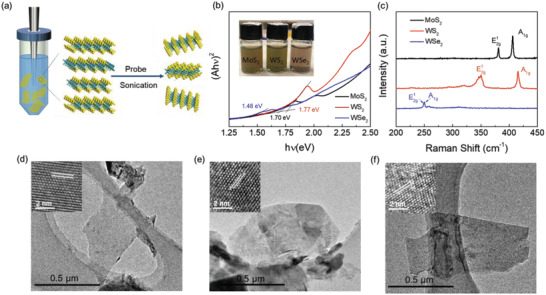
a) Schematic illustration for preparation of MoS_2_, WS_2_, and WSe_2_ flakes (*MX*_2_) via liquid phase exfoliation assisted by sonication. b) The images (shown in the inset) and Tauc plots of *MX*_2_ flakes in IPA dispersions. The Tauc plots are derived from the absorption spectra. c) Raman spectra of *MX*_2_ flakes spin‐coated on Si wafers. TEM and HRTEM images of as‐exfoliated d) MoS_2_, e) WS_2_, and f) WSe_2_, respectively.

PSCs with an inverted structure of ITO/NiO*_x_*/*MX*_2_/FASnI_3_/PCBM/BCP/Ag were fabricated. *MX*_2_ interlayers were deposited on the surface of NiO*_x_* by spin‐coating from their IPA solutions. To have a clear observation of film morphology, *MX*_2_ flakes were spin‐coated on flat Si substrates and observed under scanning electron microscopy (SEM). The as‐deposited *MX*_2_ retains a layered structure and the statistics of the flake sizes are obtained (see Figure S2, Supporting Information). The coverage of the flakes is about 30%. A FASnI_3_ perovskite layer was then fabricated on *MX*_2_/NiO*_x_* using a one‐step anti‐solvent dripping method. Top‐view SEM images of FASnI_3_ films grown on NiO*_x_* and *MX*_2_/NiO*_x_* are shown in **Figure** **2**a–d. It can be seen that the *MX*_2_ interlayers do benefit the growth of perovskite films and lead to an enlarged grain size and less grain boundaries. FASnI_3_ perovskites fabricated on MoS_2_/NiO*_x_*, WS_2_/NiO*_x_*, and WSe_2_/NiO*_x_* exhibit average grain sizes of 607, 575, and 669 nm, respectively, which are much bigger than the grain size (286 nm) of the film prepared on pristine NiO*_x_* layer (see Figure S3, Supporting Information). This result indicates the role of *MX*_2_ interlayer as a template in regulating the lateral growth of FASnI_3_ perovskite crystallites. It has been reported that the smooth and defect‐free van der Waals (vdW) face of *MX*_2_ can provide a growth template and promote perovskite film growth with enlarged grain sizes.^[^ ^34^, ^40^ ^]^ Thus, it is reasonable to observe larger FASnI_3_ perovskite grains grown on big *MX*_2_ flakes.^[^ ^34^ ^]^

**Figure 2:**
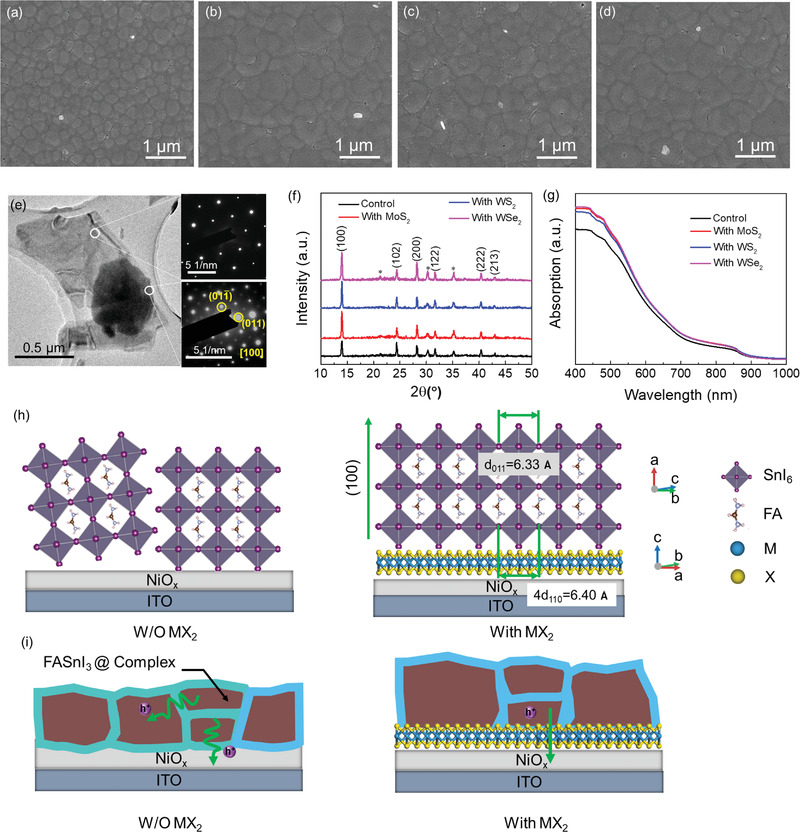
Top‐view SEM images of FASnI_3_ perovskite films fabricated on a) NiO*_x_*, b) MoS_2_/NiO*_x_*, c) WS_2_/NiO*_x_*, and d) WSe_2_/NiO*_x_*, respectively. e) TEM image of a WSe_2_ flake with a perovskite film grown on it. The SAED patterns of WSe_2_ (on the right top) and FASnI_3_ perovskite (on the right down) corresponding to the areas as indicated. f) XRD and g) UV–vis spectra of FASnI_3_ perovskite films grown on pristine and *MX*_2_‐modified NiO*_x_*/ITO. * Denote to the peaks of ITO. h) Schematic diagram of the growth of the FASnI_3_ grain on NiO*_x_* (left) and vdW epitaxial growth of the FASnI_3_ grain on a *MX*_2_ (WSe_2_) surface (right) from the side view. i) Schematic diagram for the structures of perovskite films grown on NiO*_x_* (left) and *MX*_2_ (right) surfaces.

To have a scrutiny of the crystallization and growth of FASnI_3_ perovskite grains on the surface of *MX*_2_, *MX*_2_ (i.e., WSe_2_) flakes with perovskite grains on their surfaces were observed under TEM. Figure 2e shows the image of a FASnI_3_/WSe_2_ heterojunction. The selected area electron diffraction (SAED) patterns obtained from two different parts of the heterojunction exhibit a hexagonal pattern (right top) and a square pattern (right down), respectively, corresponding to the hexagonal crystal structure of WSe_2_ and the orthorhombic structure of FASnI_3_, which confirms the overlying of a perovskite film on a WSe_2_ flake. Two diffraction spots in the square pattern are indexed to (011) and (01$\bar{1}$) planes of FASnI_3_ perovskite, and the zone axis of [100] can be derived. Such a calibration result shows favorable agreement with the simulated SAED pattern from its crystallographic information framework (CIF) file (see Figure S4, Supporting Information). Thus, WSe_2_ tends to modulate the growth of perovskite grain with a preferred (100) orientation, with (011) and (01$\bar{1}$) planes grown along the substrate.

Perovskite films on pristine NiO*_x_* and *MX*_2_/NiO*_x_* substrates were characterized by X‐ray diffraction (XRD). As shown in Figure 2f, all samples exhibit similar characteristic XRD peak positions, signifying that the *MX*_2_ interlayer did not affect the crystal structure of the upper perovskite layer. Notably, the peak intensities of (100) and (200) increase for perovskite films on *MX*_2_/NiO*_x_* substrates and the peak intensity ratio of (100)/(102) increases from 1.19 for NiO*_x_* to 2.61, 2.43, and 2.68, for MoS_2_, WS_2_, and WSe_2_ modified NiO*_x_*, respectively. Hence, perovskite films deposited on *MX*_2_/NiO*_x_* have better crystallinity and oriented growth along the (100) direction.

UV–vis absorption spectra of the perovskite films on pristine NiO*_x_* and *MX*_2_/NiO*_x_* substrates are shown in Figure 2g. Considerable absorption enhancement is observed for perovskite films with the incorporation of a *MX*_2_ interlayer within the visible wavelength region. Such enhancement is directly related to the enlarged perovskite grains and better crystallinity. UV–vis absorption spectra of NiO*_x_* substrates processed without and with *MX*_2_ interlayer were also characterized (see Figure S5, Supporting Information). The incorporation of *MX*_2_ does not change the optical transmittance, indicating little contribution of *MX*_2_ to the whole absorption of the perovskite films processed on *MX*_2_/NiO*_x_* substrates. Moreover, the slightly higher absorption of perovskite films processed on WSe_2_/NiO*_x_* than MoS_2_/NiO*_x_* and WS_2_/NiO*_x_* is consistent with the enhanced crystallinity revealed from SEM (Figure 2a–d) and XRD (Figure 2f). Based on the above results, the crystal structures and the relative orientations of WSe_2_ and FASnI_3_ are depicted in Figure 2h and Figure S6, Supporting Information. The NiO*_x_* layer composed of random round particles or clusters will not have lattice match with the perovskite and regulate its preferred orientation. WSe_2_ flakes have a smooth surface with ordered atom arrangement, where the quadruple distance of the (110) planes in WSe_2_ (4d_110_ = 6.4 Å) is close to the lattice distance of (011) in FASnI_3_ (6.33 Å) and thus perfect for the epitaxial growth of FASnI_3_ on WSe_2_ along the (100) direction.

Notably, Sn‐based perovskite grains are encapsulated with an amorphous layer made of SnF_2_, SnCl_2_, and other additives, which is a general technique that has been developed specifically for Sn‐based PSCs to prohibit the oxidation of the perovskite grains.^[^ ^20^, ^23^, ^41^ ^]^ Here, we fabricated FASnI_3_ perovskite films with SnCl_2_ and gallic acid (GA) coadditives, which the enables capping of the perovskite grains with an amorphous SnCl_2_—GA complex layer as described in our previous report.^[^ ^23^ ^]^ However, the complex restricts charge transfer from the grains to the HTL due to its low conductivity. As shown in Figure 2i, the direct epitaxial growth of a perovskite film on WSe_2_ flakes can be favorable for charge transfer from perovskite to the underlying NiO*_x_* HTL. The perfect interface between perovskite and WSe_2_ flakes will enable charge transfer without the influence of the complex layer, which is expected to result in improved device performance.

The potential of using exfoliated *MX*_2_ flakes as an interlayer in PSCs is further evaluated. Considering that the implementation of *MX*_2_ on NiO*_x_* HTL can exert impacts on charge transfer between HTL/perovskite interface,^[^ ^42^ ^]^ we adjusted their spin‐coating times and investigated the corresponding PSC performances. The device performances and PV parameters of the devices with MoS_2_, WS_2_, and WSe_2_ spin‐coated for different times can be found in Figure S7 and Table S1, Supporting Information. **Figure** **3**a shows representative photocurrent density–voltage (J–V) curves of the three *MX*_2_‐modified devices at optimum conditions. The control device exhibits a PCE of 8.03%, a *J*_sc_ of 19.44 mA cm^−2^, a *V*_oc_ of 0.59 V, and a FF of 70.0%. The incorporation of MoS_2_ increases the PCE to 8.52%, as a result of the significantly enhanced *J*_sc_ to 21.75 mA cm^−2^, although its *V*_oc_ is slightly decreased to 0.57 V. Replacing MoS_2_ by WS_2_ further increases the PCE to 8.75%, with a *V*_oc_ of 0.58V, a *J*_sc_ of 21.44 mA cm^−2^, and a FF of 70.4%. The best PCE of 9.90% is achieved for WSe_2_ incorporated device, with a considerably improved *V*_oc_ (0.63 V), enhanced *J*_sc_ (21.82 mA cm^−2^), and FF (72.0%). Therefore, all *MX*_2_ incorporated devices show improved *J*_sc_ than the control device, which can be attributed to enhanced light absorption of perovskites on the 2D *MX*_2_ flakes. Notably, the *J*_sc_ differences among *MX*_2_ incorporated devices are not obvious, whereas the *V*_oc_ variation is quite noticeable.

**Figure 3:**
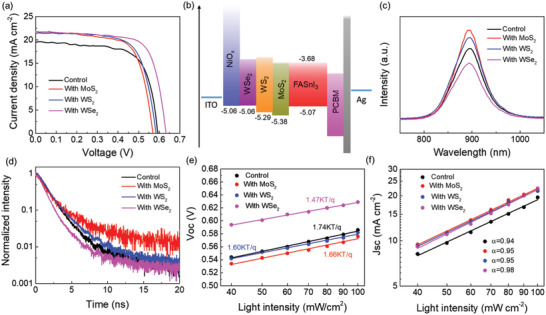
a) Representative *J–V* curves of *MX*_2_ incorporated devices at the optimum condition. b) Energy level diagram of the PSC with incorporation of *MX*_2_. c) PL and d) TRPL spectra of FASnI_3_ perovskite films fabricated on pristine NiO*_x_*/ITO and NiO*_x_*/ITO substrates modified with *MX*_2_. Light intensity dependence of e) *V*_oc_ and f) *J*_sc_ of the control and *MX*_2_ incorporated devices.

Ultraviolet photoelectron spectroscopy (UPS) measurements were further conducted to investigate the energy levels of the FASnI_3_ layer and NiO*_x_* film without and with *MX*_2_ modification, as shown in Figures S8 and S9, Supporting Information. Their VBMs are calculated to be −5.07 (FASnI_3_), −5.06 (NiO*_x_*), −5.38 (MoS_2_), −5.29 (WS_2_), and −5.06 eV(WSe_2_) according to the following equation:^[^ ^43^ ^]^

(1) $$E_{\mathrm{VB}}=E_{\mathrm{cutoff}}\;-E_{F}-h\nu \;\left(h\nu \;=\;21.22\;eV\right)$$

where *E*_cutoff_ represents the secondary electron cutoff value and *E*_F_ represents the Fermi level. The corresponding conduction band minimum (CBM) can be further derived from *E*_CB_ = *E*_VB_ + *E*_g_, where *E*_g_ is obtained from the absorption edge (see Figures 1b and 2g). Thus the energy level diagram of the device is depicted in Figure 3b. MoS_2_ and WS_2_ have lower VBM than FASnI_3_ perovskite, whereas WSe_2_ shows good VBM alignment in the NiO*_x_*/WSe_2_/perovskite/PCBM multilayer structure. Thus, the incorporation of WSe_2_ favors charge transfer and collection at the NiO*_x_*/perovskite interface and has less voltage loss, which is expected to induce higher device *V*_oc_ than MoS_2_ and WS_2_.

To further investigate the effect of *MX*_2_ interlayer on the charge dynamic process at the interface of NiO*_x_*/perovskite, steady state photoluminescence (PL) and time‐resolved photoluminescence (TRPL) were characterized on FASnI_3_ films fabricated on *MX*_2_/NiO*_x_*/glass substrates. As shown in Figure 3c, the samples with the incorporation of MoS_2_ and WS_2_ exhibit higher PL intensity than the control sample, as a result from the better film quality of the former perovskite films. However, the incorporation of WSe_2_ weakens the PL intensity, which can only be accounted for by that the WSe_2_ bridges the energy levels between perovskite film and NiO*_x_* layer well and therefore boosts charge transfer across the interface. The TRPL results of the corresponding samples are shown in Figure 3d. The TRPL spectra are assumed in first order decay and fitted by a single‐exponential function of Y = A*exp(−t/*τ*) + y0, where *A* and *τ* represent the amplitude and PL decay lifetime respectively. The FASnI_3_ film fabricated on NiO*_x_* exhibits a characteristic TRPL lifetime of 1.4 ns. With MoS_2_ and WS_2,_ the PL decay lifetimes are prolonged to 1.55 and 1.43 ns, respectively. A faster PL quenching (lifetime of 1.3 ns) is observed for the sample with WSe_2_, indicating that WSe_2_ is more effective for charge transfer than the pristine NiO*_x_* as well as MoS_2_ and WS_2_ modified NiO*_x_* layers. Therefore, WSe_2_ is most favorable for the collection of photo‐generated carriers by NiO*_x_* and consequently reduces interfacial charge recombination loss.

We also conducted light intensity (*P*_light_) dependent *J–V* measurements of the devices to examine the effect of *MX*_2_ interlayer on charge recombination. The dependence of *V*_oc_ on *P*_light_ is plotted according to the relation: *V*_oc_∝*n*k*T*/*q* ln(*P*_light_), where *n*, k, *T*, and *q* are ideality factor, Boltzmann constant, temperature in K, and elementary charge, respectively. The value *n* reflects the carrier traps assisted recombination in the active layer or at interfaces at open circuit condition.^[^ ^44^, ^45^ ^]^ As shown in Figure 3e, the control device shows a slope of *V*_oc_ versus semilogarithmic *P*_light_ of 1.74 kT q^−1^, which is only slightly reduced to 1.66 kT q^−1^ for an MoS_2_ incorporated device and 1.60 kT q^−1^ for a WS_2_ incorporated device. Notably, the lowest slope of 1.47 kT q^−1^ is observed for a WSe_2_ incorporated device, indicating that the trap‐assisted recombination losses are greatly suppressed than in other devices. The less trap induced recombination in a WSe_2_ incorporated device benefits from its proper energy level alignment with NiO*_x_* and FASnI_3_, which promotes hole transfer from perovskite to HTL.

Figure 3f plots the dependence of *J*_sc_ on *P*_light_ on a log–log scale, according to the equation: *J*_sc_∝(*P*_light_)^*α*^_._ The exponent *α* is close to 1 when all carriers transfer to the electrodes without bimolecular recombination.^[^ ^46^ ^]^ WSe_2_‐incorporated device shows an *α* value of 0.98, higher than 0.94 for the control device and 0.95 for both MoS_2_ and WS_2_ incorporated devices. The results indicate that the WSe_2_ interlayer can effectively enhance the charge transportation property and reduce the bimolecular recombination.

The effect of WSe_2_ interlayer on hole transport at the NiO*_x_*/perovskite interface is further characterized by measuring the space‐charge limited current using hole‐only devices (ITO/NiO*_x_*/without or with WSe_2_/FASnI_3_/P3HT/Au). It can be seen from Figure S10, Supporting Information, that the devices without and with WSe_2_ incorporation show the trap filled limit voltage (*V*_TFL_) of 2.97 and 2.19 V, corresponding to the trap density (*N*_t_) of 4.69 × 10^16^ and 3.45 × 10^16^ cm^−3^, respectively. The lower trap density for the latter signifies that the hole trap is effectively passivated by WSe_2_ at the NiO*_x_*/perovskite interface, which facilitates hole transfer at such an interface with a lower trapping possibility.

**Figure** **4**a presents the *J–V* curves of the best‐performing WSe_2_ incorporated device under AM 1.5 one‐sun illumination. It displays a *V*_oc_ of 0.63 V, a *J*_sc_ of 22.71 mA cm^−2^, an FF of 73.2% and results in a PCE of 10.47% for reverse scan; and a *V*_oc_ of 0.64 V, a *J*_sc_ of 22.14 mA cm^−2^, an FF of 71.0%, and a PCE of 10.06% for forward scan. The negligible hysteresis proves the high quality of the perovskite film with suppressed ion migration as well as efficient interfacial charge transport. Figure 4b shows the external quantum efficiency (EQE) spectrum for the device. *J*_sc_ is integrated to be 22.3 mA cm^−2^, which is close to the value as observed in *J–V* curves. The stable output of ≈10.2% is acquired at the maximum power point, as shown in Figure 4c. Moreover, the histogram of the PCEs of 25 WSe_2_‐incorporated devices is presented in Figure 4d. The narrow distribution of the PCE data further illustrates the reliability and reproducibility of our devices. Long term stability of the WSe_2_‐incorporated device stored in air (relative humidity ≈20%) without encapsulation was monitored and shown in Figure S11, Supporting Information. It shows a high stability, retaining about 82% of the initial PCE after 1000 h.

**Figure 4:**
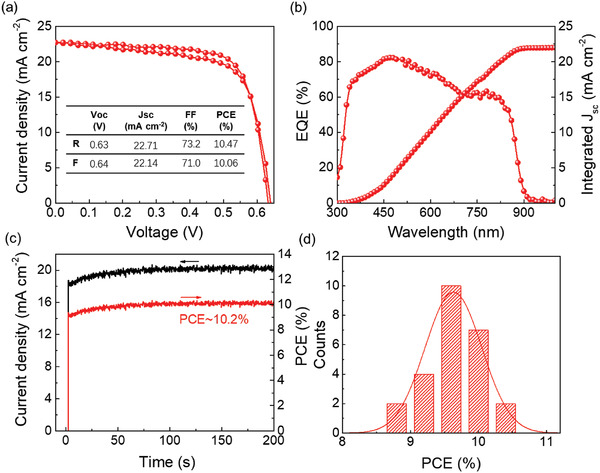
a) *J–V* curves for the champion WSe_2_ incorporated device in forward and reverse scans. b) EQE and integrated *J*_sc_ curves for the champion PSC with incorporation of WSe_2_. c) Stable photocurrent density and PCE of a WSe_2_ incorporated device measured at the maximum power point. d) The statistics of PCEs of WSe_2_ incorporated PSCs.

In summary, we reported for the first time the introduction of liquid exfoliated *MX*_2_ flakes as an interlayer between NiO*_x_*/FASnI_3_ perovskite to enhance the performance of inverted Sn‐based PSCs. The *MX*_2_ flakes with smooth surfaces act as a growth template that can lead to the vdW epitaxial growth of FASnI_3_ perovskite grains with enlarged size and preferred orientation. More importantly, we find that the band structure of the 2D flakes is critical to the charge transfer in the resultant devices. In comparison with MoS_2_ and WS_2_, WSe_2_ possesses a higher VBM and is judiciously selected as an efficient charge transport interlayer due to its high hole mobility and proper energy alignment with the VBMs of HTL and perovskite film. The resultant WSe_2_ incorporated PSCs exhibit increased photovoltaic parameters in *J*_sc_, *V*_oc_, and FF, which is due to the synergy of enhanced charge transport, lesser interfacial recombination, and stronger light absorption. Eventually, a PCE of 10.47% is obtained for the champion WSe_2_‐incorporated device, which is among the highest efficiency for FASnI_3_‐based PSCs (Table S2, Supporting Information). This work provides a convenient strategy to control the growth of FASnI_3_ perovskite film and optimize its interface property, which is also suitable for the preparation of other Sn‐based perovskite optoelectronic devices.^[^ ^47^, ^48^ ^]^

## Experimental Section

##### Materials

Formamidinium iodide (FAI) was purchased from GreatCell Solar. Nickel(II) nitrate hexahydrate (Ni(NO_3_)_2_ 6H_2_O, 98%) and SnI_2_ (99.999%) were purchased from Alfa Aesar. Gallic acid (99%) and SnCl_2_ (99.99%) were purchased from Aladdin. Bathocuproine (BCP) was purchased from Sigma‐Aldrich. Phenyl‐C71‐butyric acid methyl ester (PCBM) was purchased from Nano‐C.

##### Preparation of *MX*_2_ Flakes

100 mg of bulk MoS_2_, WS_2_, and WSe_2_ materials were carefully grinded into fine powders and then dispersed in 10 ml isopropanol in a centrifuge tube, respectively. The dispersion was further sealed with Parafilm and sonicated with a probe sonic tip for 12 h. The temperature of tube was kept at 5 °C under an ice bath. After that, the dispersion was centrifuged at 2000 rpm for 5 min to extract the unexfoliated materials and the supernatant containing exfoliated flakes was taken for further use.

##### Device Fabrication

ITO glass substrates were sequentially washed by acetone, deionized water, and isopropanol. NiO*_x_* solution (6.5 mg mL^−1^ in DI water, synthesis of NiO*_x_* powder can be referred to our previous report) was spin‐coated on ITO substrates at 4000 rpm for 30 s then annealed at 150 *°*C for 20 min. For NiO*_x_* modification, the as‐prepared *MX*_2_ flakes in IPA solution were repeatedly spin‐coated on it to achieve the desired amount. 0.8 m FASnI_3_ perovskite precursor solution containing FAI (1 mmol), SnI_2_ (1 mmol), SnCl_2_ (0.07 mmol), and gallic acid (0.01 mmol) in mixed DMF/DMSO solution (v/v = 10:1) was spin‐coated on the substrates at 5000 rpm for 30 s and chlorobenzene was dripped at 13 s after starting. The as‐spun perovskite films were annealed at 70 *°*C for 5 min. Afterwards, PCBM (20 mg mL^−1^ in chlorobenzene) was spin‐coated at 1500 rpm for 30 s, followed by spin‐coating BCP (0.5 mg mL^−1^ in IPA) at 5000 rpm for 30 s. The devices were completed by thermal evaporation of 100 nm Ag on the BCP layer.

##### Characterizations

*J–V* curves of PSCs were measured using a Keithley 2400 source meter under the illumination of AM 1.5G, 100 mW cm^−2^ solar simulator (Newport 91160, 300 W). The EQE of the PSCs was measured using an EQE system equipped with a xenon lamp (Oriel 66902), an Si detector (Oriel 76175_71 580), a monochromator (Newport 66902), and a dual channel power meter (Newport 2931_C). UV–vis spectra were measured with a Perkin Elmer_UV–vis‐NIR spectrometer. SEM images were characterized by a field emission scanning electron microscope (Tescan MAIA3). XRD measurements were performed on a Rigaku Smartlab Diffractometer. Ultraviolet photoelectron spectroscopy (UPS) was measured on Thermo Fisher Scientific system. Steady‐state photoluminescence (PL) and time‐resolved photoluminescence (TRPL) were measured using an Edinburgh FLS920 fluorescence spectrophotometer. The Raman measurements were performed on a WITEC_Confocal Raman system. Field emission TEM was performed with a JEOL Model JEM‐2100F instrument operated at 200 kV. Atomic force microscopy (AFM) images were collected using Bruker NanoScope 8.

## Conflict of Interest

The authors declare no conflict of interest.

## Supporting information

Supporting Information

## Data Availability

The data that support the findings of this study are available from the corresponding author upon reasonable request.
