# Supplementary material for: 2D WSe2 Flakes for Synergistic Modulation of Grain Growth and Charge Transfer in Tin‐Based Perovskite Solar Cells
Source: Adv Sci (Weinh). 2021 Mar 27;8(11):2004315. doi: 10.1002/advs.202004315 (PMC8188186; doi:10.1002/advs.202004315)
Supplement: Supplementary file 1 — Supporting Information [file ADVS-8-2004315-s001.pdf]

WILEY-VCH

© 2021 Wiley-VCH GmbH

ADVANCED SCIENCE
Open Access

Supporting Information

for *Adv. Sci.*, DOI: 10.1002/advs.202004315

2D WSe_2 Flakes for Synergistic Modulation of Grain Growth and Charge Transfer in Tin-based Perovskite Solar Cells

*Tianyue Wang, Fangyuan Zheng, Guanqi Tang, Jiupeng Cao, Peng You, Jiong Zhao, and Prof. Feng Yan**

Supporting Information

2D WSe_2 flakes for synergistic modulation of grain growth and charge transfer in tin-based perovskite solar cells

*Tianyue Wang, Fangyuan Zheng, Guanqi Tang, Jiupeng Cao, Peng You, Jiong Zhao, and Feng Yan**

T. Y. Wang, F. Y. Zheng, G. Q. Tang, J. P. Cao, P. You, J. Zhao, Prof. F. Yan
Department of Applied Physics, The Hong Kong Polytechnic University, Hung Hom, Kowloon, Hong Kong
E-mail: apafyan@polyu.edu.hk

Figures:

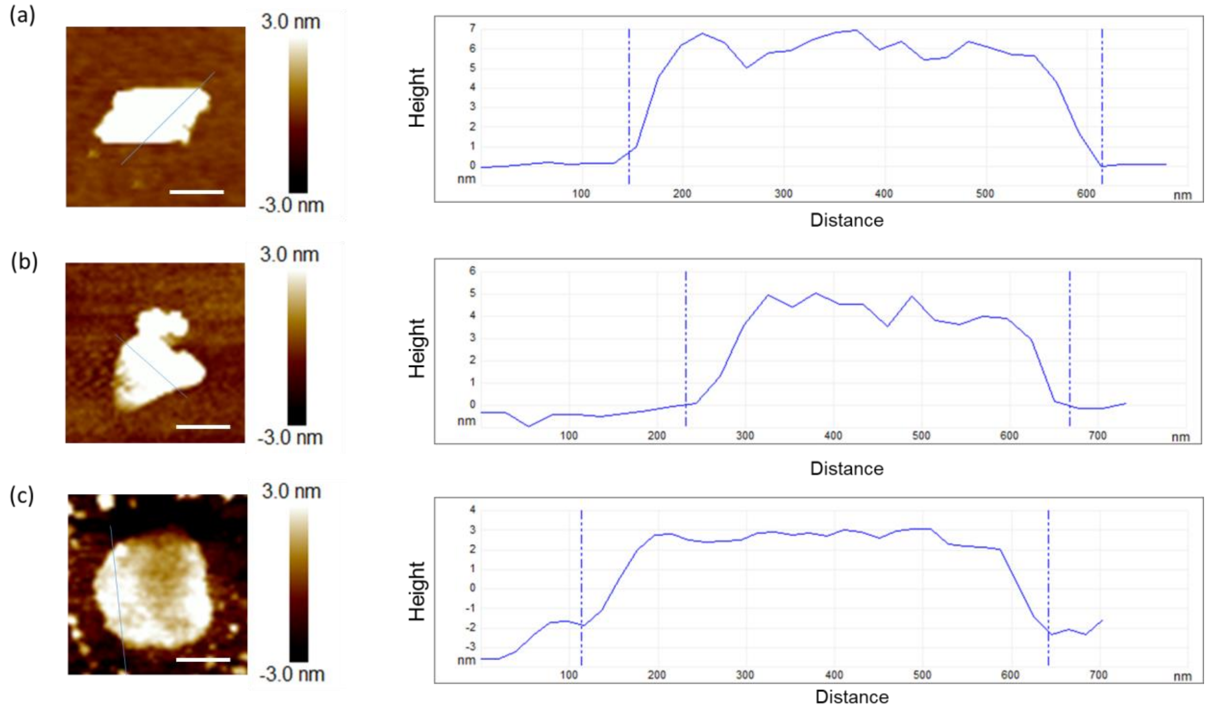

Figure S1 Representative AFM images of a) MoS_2, b) WS_2 and c) WSe_2 flakes deposited on Si substrates. Scale bar: 300 nm. The height profiles on the right were extracted from the indicated blue lines in the AFM images.

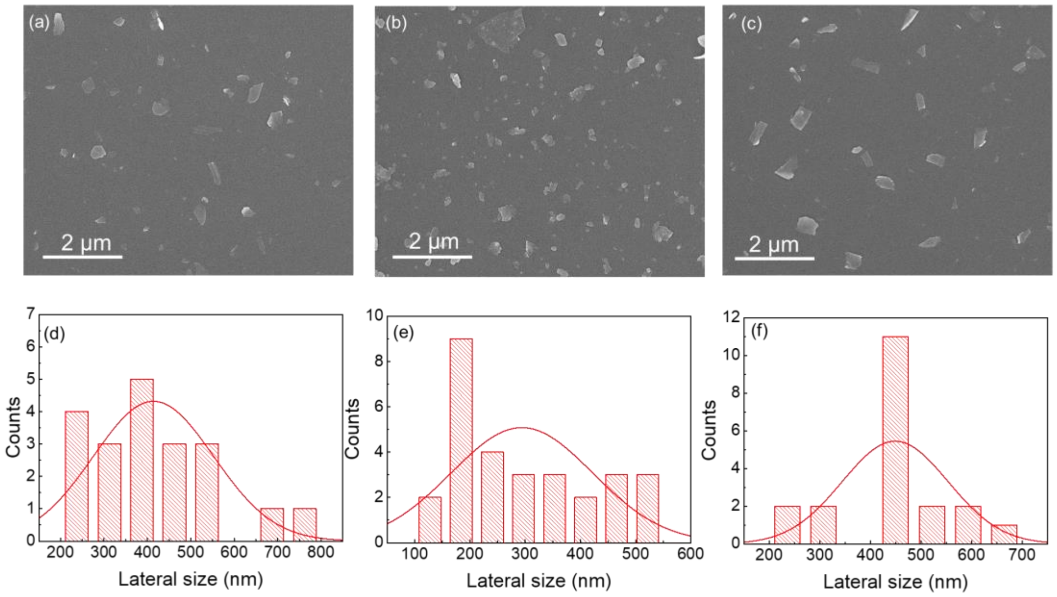

Figure S2 SEM images of as-exfoliated a) MoS_2, b) WS_2 and c) WSe_2 flakes spin-coated on Si-substrates. Lateral size distributions of d) MoS_2, e) WS_2 and f) WSe_2 flakes extracted from a-c).

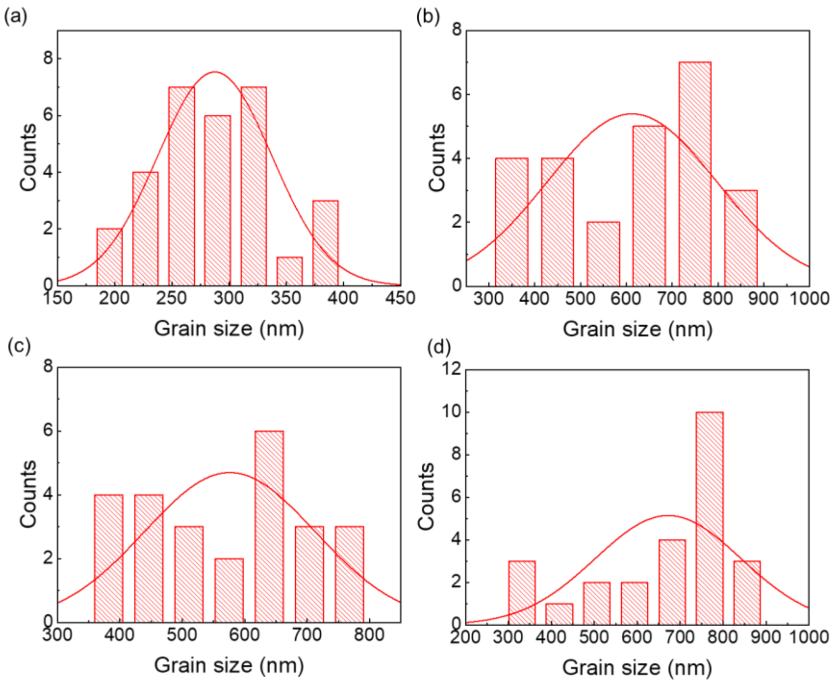

Figure S3 Statistical grain size distribution of $FASnI_3$ films deposited on a) NiO_x/ITO, as well as b) MoS_2, c) WS_2, and d) WSe_2 modified NiO_x/ITO.

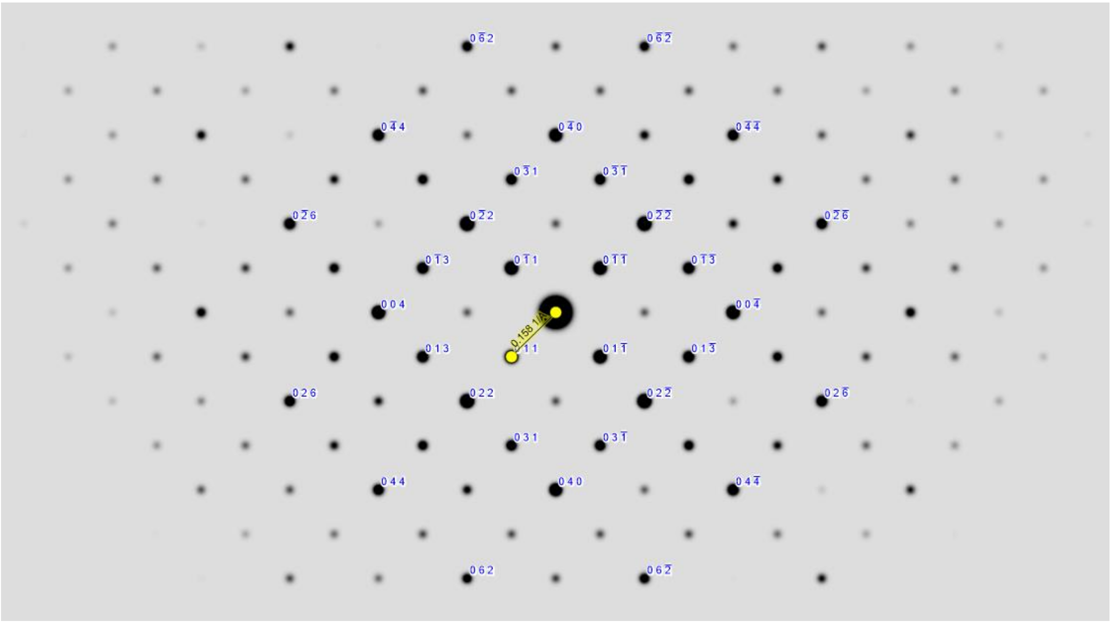

Figure S4 Simulated transmission electron diffraction of $FASnI_3$ single crystal from view direction of indices [100]. The simulation was performed by CrystalMaker and SingleCrystal Softwares according to the CIF of $FASnI_3$ (orthorhombic Amm2 space group).

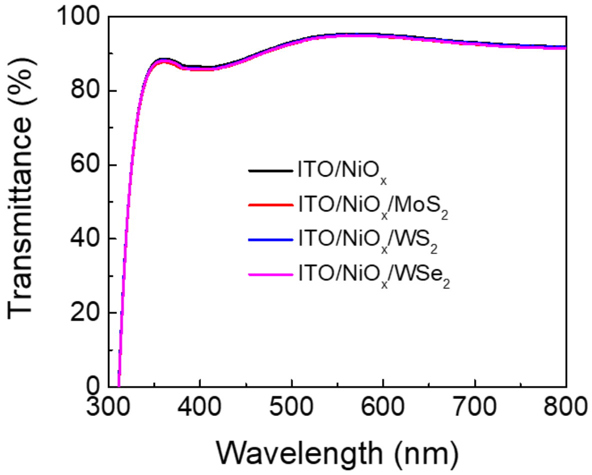

Figure S5 Transmittance spectra of NiO_x/ITO/glass substrates processed w/o and with MX_2 interlayer.

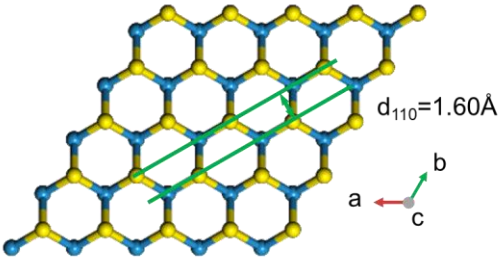

Figure S6 Atomic crystal structure of the (110) plane of WSe_2 from the top view.

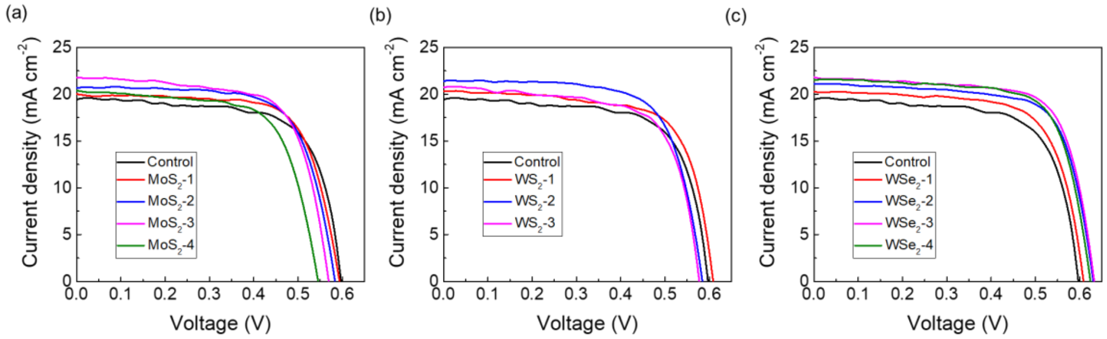

Figure S7 Typical J-V curves of the control PSC and MX_2- incorporated PSCs prepared by spin-coating MX_2 dispersions for different times.

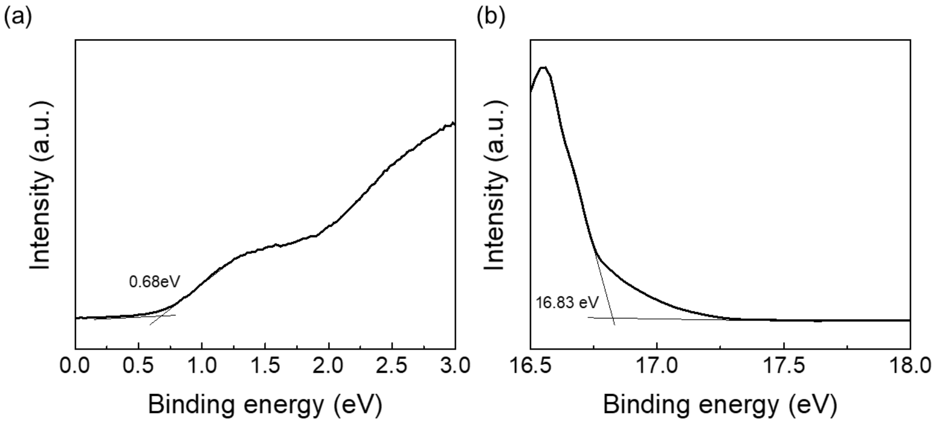

Figure S8 a) Near E_f region and b) the secondary electron cutoff region of UPS spectra of the $FASnI_3$ perovskite film.

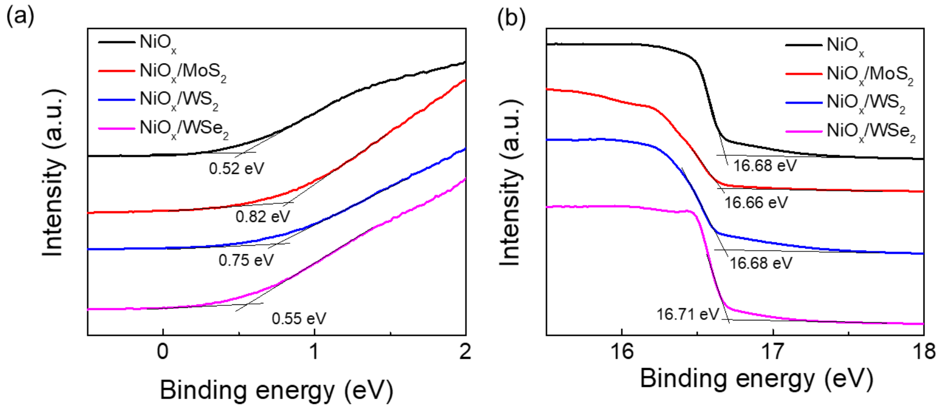

Figure S9 a) Near E_f region and b) the secondary electron cutoff region of UPS spectra of the NiO_x layer and MX_2 modified NiO_x layers.

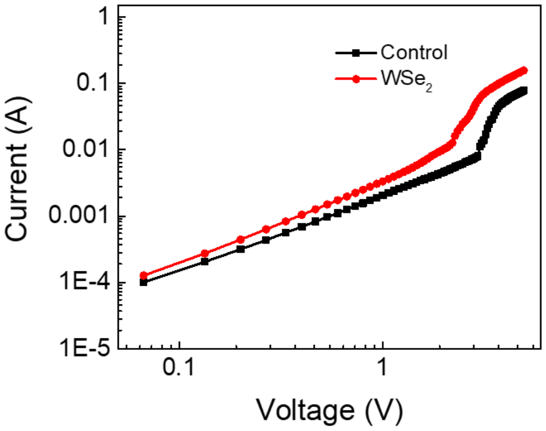

Figure S10 Dark I-V curves of the hole-only devices w/o and with WSe_2 incorporation.

Note: Hole-only devices with a structure of ITO/NiO_x/w/o or with WSe_2/$FASnI_3$/P3HT/Au were used to measure the dark I-V curves. The trap density (N_t) in the perovskite film can be calculated by the equation:

$$N_t = \frac{2\varepsilon\varepsilon_0 V_{TFL}}{qL^2}$$

where V_{TFL} is the trap filled limit voltage, ε is the relative dielectric constant of $FASnI_3$ (~5.7), ε_0 is the vacuum permittivity, q is the electron charge, and L is the perovskite film thickness.

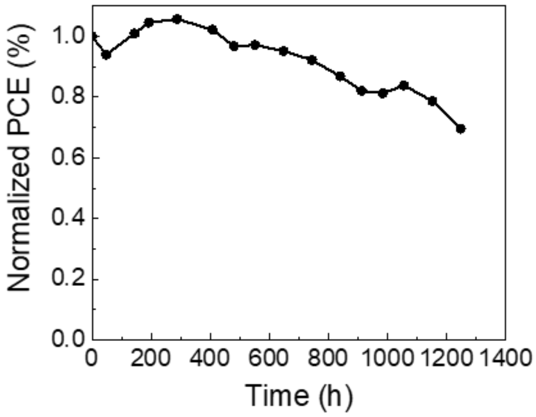

Figure S11 Long-term stability of the WSe_2 incorporated PSC (unencapsulated) when stored in the air with a relative humidity (RH) about 20% and measured in the ambient condition (RH≈45%).

Tables:

Table S1 Photovoltaic parameters of MX_2 processed PSCs (MX_2 dispersions were spin-coated for different times) extracted from Figure S7.

	V_{OC} (v)	J_{SC} (mA cm^{-2})	FF (%)	PCE (%)
**Control**	0.59	19.44	70.0	8.03
**MoS_2-1**	0.59	20.02	71.0	8.39
**MoS_2-2**	0.58	20.67	70.4	8.44
**MoS_2-3**	0.57	21.75	68.7	8.52
**MoS_2-4**	0.54	20.40	67.8	7.47
**WS_2-1**	0.61	20.32	69.8	8.65
**WS_2-2**	0.58	21.44	70.4	8.75
**WS_2-3**	0.58	20.71	69.9	8.39
**WSe_2-1**	0.61	20.27	70.0	8.66
**WSe_2-2**	0.63	21.14	71.9	9.56
**WSe_2-3**	0.63	21.82	72.0	9.90
**WSe_2-4**	0.62	21.54	72.4	9.67

Table S2 Efficiencies of $FASnI_3$ –based PSCs in literature.

**Composition**	**Device structure**	**PCE (%)**	**Ref**
$FASnI_3$ (SnF_2)	FTO/c-TiO_2/mp-TiO_2/perovskite/spiro-OMeTAD/Au	2.1	[1]
$FASnI_3$ (SnF_2, N_2H_5Cl)	ITO/PEDOT:PSS/perovskite/PCBM/BCP/Ag	5.4	[2]
$FASnI_3$ (SnF_2)	ITO/PEDOT:PSS/perovskite/C_{60}/BCP/Ag	6.22	[3]
$FASnI_3$ (SnF_2, TMA)	ITO/PEDOT:PSS/perovskite/C_{60}/ bis-C_{60}/Ag	7.09	[4]
$FASnI_3$ (SnF_2, pyrazine)	FTO/c-TiO_2/mp-TiO_2/perovskite/spiro-OMeTAD/Au	4.8	[5]
$FASnI_3$ (SnF_2, $EDAI_2$)	ITO/PEDOT:PSS/ perovskite/C_{60}/BCP/Ag	8.9	[6]
$FASnI_3$ (SnF_2, PTN-Br)	ITO/PEDOT:PSS/perovskite/C_{60}/BCP/Ag	7.94	[7]
$FASnI_3$ (SnF_2,FOEI)	ITO/PEDOT:PSS/ perovskite/C_{60}/BCP/Ag	10.16	[8]
$FASnI_3$ (SnF_2, $EDAP_2$)	ITO/PEDOT:PSS/ perovskite/C_{60}/BCP/Ag	6.8	[9]
$FASnI_3$ (SnF_2, PHCl)	ITO/PEDOT:PSS/ perovskite/C_{60}/BCP/Ag	11.4	[10]
$FASnI_3$ (SnF_2, PAI)	ITO/PEDOT:PSS/ perovskite/C_{60}/BCP/Ag	11.22	[11]
$FASnI_3$ ($SnCl_2$,GA)	ITO/NiO_x/WSe_2/perovskite/PCBM/BCP/Ag	10.47	This work

References:

[1] T. M. Koh, T. Krishnamoorthy, N. Yantara, C. Shi, W. L. Leong, P. P. Boix, A. C. Grimsdale, S. G. Mhaisalkar, N. Mathews, *J. Mater. Chem. A* **2015**, *3*, 14996.

[2] M. E. Kayesh, T. H. Chowdhury, K. Matsuishi, R. Kaneko, S. Kazaoui, J.-J. Lee, T. Noda, A. Islam, *ACS Energy Lett.* **2018**, *3*, 1584.

[3] W. Liao, D. Zhao, Y. Yu, C. R. Grice, C. Wang, A. J. Cimaroli, P. Schulz, W. Meng, K. Zhu, R. G. Xiong, Y. Yan, *Adv Mater* **2016**, *28*, 9333.

[4] Z. Zhu, C. C. Chueh, N. Li, C. Mao, A. K. Y. Jen, *Adv. Mater.* **2018**, *30*, 1703800.

[5] S. J. Lee, S. S. Shin, Y. C. Kim, D. Kim, T. K. Ahn, J. H. Noh, J. Seo, S. I. Seok, *J. Am. Chem. Soc.* **2016**, *138*, 3974.
[6] E. Jokar, C.-H. Chien, A. Fathi, M. Rameez, Y.-H. Chang, E. W.-G. Diau, *Energy Environ. Sci.* **2018**, *11*, 2353.
[7] C. Liu, J. Tu, X. Hu, Z. Huang, X. Meng, J. Yang, X. Duan, L. Tan, Z. Li, Y. Chen, *Adv. Funct. Mater.* **2019**, *29*, 1808059.
[8] X. Meng, Y. Wang, J. Lin, X. Liu, X. He, J. Barbaud, T. Wu, T. Noda, X. Yang, L. Han, *Joule* **2020**, *4*, 902.
[9] S. Shahbazi, M.-Y. Li, A. Fathi, E. W.-G. Diau, *ACS Energy Lett.* **2020**, *5*, 2508.
[10] C. Wang, F. Gu, Z. Zhao, H. Rao, Y. Qiu, Z. Cai, G. Zhan, X. Li, B. Sun, X. Yu, B. Zhao, Z. Liu, Z. Bian, C. Huang, *Adv. Mater.* **2020**, *32*, 1907623.
[11] X. Liu, T. Wu, J.-Y. Chen, X. Meng, X. He, T. Noda, H. Chen, X. Yang, H. Segawa, Y. Wang, L. Han, *Energy Environ. Sci.* **2020**, *13*, 2896.
